# Supplementary material for: The Intriguing Biogeographic Pattern of the Italian Wall Lizard Podarcis siculus (Squamata: Lacertidae) in the Tuscan Archipelago Reveals the Existence of a New Ancient Insular Clade
Source: Animals (Basel). 2023 Jan 23;13(3):386. doi: 10.3390/ani13030386 (PMC9913114; doi:10.3390/ani13030386)
Supplement: Supplementary file 1 [file animals-13-00386-s001.zip › supplementary.pdf]

Table S2. Absolute phenotypes frequencies in males.

Location	Campestris	Calabresiae	Intermediate1	Reticulated	Intermediate2	Concolor
Continent	155	2	0	0	0	0
Elba	14	0	0	0	0	0
Capraia	7	0	9	16	0	2
Cerboli	13	0	0	0	0	0
Montecristo	0	41	1	1	0	0
Giglio	0	0	5	38	20	31
Giannutri	6	5	9	23	0	15

Table S3. Absolute phenotypes frequencies in females.

Location	Campestris	Calabresiae	Intermediate1	Reticulated	Intermediate2	Concolor
Continent	102	0	0	0	0	0
Elba	10	0	0	0	0	0
Capraia	11	0	1	0	0	0
Cerboli	6	0	0	0	0	0
Montecristo	0	18	0	0	0	0
Giglio	3	1	12	14	14	19
Giannutri	5	4	0	6	5	6

Table S4. Primers for microsatellites loci used in genetic analyses.

Locus	Multiplex	Primer sequence	Ta (°C)	Allele range	N° alleles	References
Pb73	Mix1	**FAM**-GCCCATGTCACTTCAGGTAGAAGC<br>GAAAACTAGGAGTTAGGGAGAAGG	57	126-134	5(2)	Pinho et al., 2004
C9	Mix1	**VIC**-CATTGCTGGTTCTGGAGAAAG<br>CCTGATGAAGGGAAGTGGTG	58	126-159	10(3)	Nembini and Opplinger, 2003
Pli24	Mix2	**FAM**-CCACAAGGACTCAGGCTCTC<br>TCCCCCACTTAAGCATGTTC	56	101-113	4	Bloor et al., 2011
Pli4	Mix2	**FAM**-TCAGTTCATGCATAAGGTCCA<br>TTCGGCATTTTTCTTCAGGT	56	319-439	24(9)	Bloor et al., 2011
Pli18	Mix2	**NED**-CAAGAATTGAGTTTGCAGTTCC<br>TGTCTGACAGAATGTGCTTCTC	56	116-168	14(2)	Bloor et al., 2011
Pb10	Mix2	**VIC**-AGTGGAATCGGCTGCAATAC<br>ACCAGTCCCAGGAATTTAGG	58	202-226	6	Pinho et al., 2004
Lv-4-a	Mix3	**PET**-CTGCAGGGAACAGAATTAACC<br>CTGCCCAGAAAGCATTTCC	60	95-121	10(2)	Boudjemadi et al., 1999
Lv-4-19	Mix3	**NED-**CTGTTGCTATTTTGTATGCTTAC<br>CTGCCCAGAAAGCATTTCC	57	114-140	10(5)	Boudjemadi et al., 1999
